# Microbial Fuel Cell-Based Biosensor for Simultaneous Test of Sodium Acetate and Glucose in a Mixed Solution

**DOI:** 10.3390/ijerph191912297

**Published:** 2022-09-28

**Authors:** Song Qiu, Luyang Wang, Yimei Zhang, Yingjie Yu

**Affiliations:** 1 College of Life Science and Technology, Beijing University of Chemical Technology, Beijing 100029, China; 2 College of Chemistry, Beijing University of Chemical Technology, Beijing 100029, China; 3 MOE Key Laboratory of Resources and Environmental System Optimization, College of Environmental Science and Engineering, North China Electric Power University, Beijing 102206, China; 4 State Key Laboratory of Organic-Inorganic Composites, Beijing University of Chemical Technology, Beijing 100029, China

**Keywords:** microbial fuel cell sensor, sodium acetate, glucose, simultaneous test

## Abstract

Most microbial fuel cell (MFC) sensors only focus on the detection of mixed solutions with respect to the chemical oxygen demand (COD) or toxicity; however, the concentrations of the individual analytes in a mixed solution have rarely been studied. Herein, we developed two types of MFC sensors, adapted with sodium acetate (MFC-A) and glucose (MFC-B) as organic substrates in the startup period. An evident difference in the sensor sensitivities (the slope value of the linear-regression curve) was observed between MFC-A and MFC-B. MFC-A exhibited a superior performance compared with MFC-B in the detection of sodium acetate (4868.9 vs. 2202 mV/(g/L), respectively) and glucose (3895.5 vs. 3192.9 mV/(g/L), respectively). To further compare these two MFC sensors, the electrochemical performances were evaluated, and MFC-A exhibited a higher output voltage and power density (593.76 mV and 129.81 ± 4.10 mW/m^2^, respectively) than MFC-B (484.08 mV and 116.21 ± 1.81 mW/m^2^, respectively). Confocal laser scanning microscopy (CLSM) and microbial-community analysis were also performed, and the results showed a richer anode biomass of MFC-A in comparison with MFC-B. By utilizing the different sensitivities of the two MFC sensors towards sodium acetate and glucose, we proposed and verified a novel method for a simultaneous test on the individual concentrations of sodium acetate and glucose in a mixed solution. Linear equations of the two variables (concentrations of sodium acetate and glucose) were formulated. The linear equations were solved according to the output voltages of the two MFC sensors, and the solutions showed a satisfactory accuracy with regard to sodium acetate and glucose (relative error less than 20%).

## 1. Introduction

Microbial fuel cells (MFCs) are novel devices that are designed according to the utilization of electrogenic bacteria, which can generate electricity by the metabolization of organic waste [1,2]. Previous research on MFCs has mainly focused on wastewater treatment coupled with energy production, power supply systems for low power devices, and biosensors [3,4,5]. However, the applications of MFCs on wastewater treatment and the power supply were only at the laboratory scale due to limitations such as the catalytic materials, operation conditions, and the poor output power [6]. Due to the characteristics of a rapid response, reliable accuracy, low cost, and high specificity, MFCs are regarded as satisfactory biosensors and are widely applied to measure a broad spectrum of environmental parameters, such as the biochemical oxygen demand (BOD), chemical oxygen demand (COD), toxicity, etc. [7,8,9,10,11].

The mechanism of an MFC sensor is based on the interactions between the electroactive bacteria and analytes (organic substrates or toxic metals), which could either increase or decrease the output signal of an MFC sensor [12]. On the one hand, organic components (namely, COD and BOD) could lead to an enhanced output signal when added to the anode chamber of an MFC sensor [13,14]. On the other hand, toxic components, such as heavy metals, formaldehyde, and antibiotics, could hinder the metabolic activity of the anode biofilm and lead to a decreased output signal during the operation of the MFC sensor [15,16,17]. The first glucose biosensor was developed 60 years ago, after which a myriad of uses of this novel biosensor have been proposed and presented with the combination of microbiological and electroanalytical expertise [18]. According to the recent literature, the research on MFC sensors has mainly focused either on testing composite items, such as the COD or toxicity, or on an individual analyte (acetate, dissolved oxygen, pH, phenol, etc.) [18]. Moreover, the emphasis of these works has been placed on the improvement in the MFC sensor performances in terms of the response time, sensibility, selectivity, and detection range by the modification of the electrode materials, the optimization of the operation conditions, the innovation of configurations, and the selection of electronic signals [19,20,21,22,23]. However, MFC sensors usually confront challenges of impaired accuracy due to fluctuations in the composition of the solution, or signal interferences between different analytes in practical application. Thus, the detection credibility is hard to guarantee. This phenomenon is sometimes prone to be ignored in the application and thus causes a deviation from the real value of the measured samples. Research has demonstrated that the addition of sodium acetate could moderate the inhibition ratio of MFC from copper (II), but the sensitivity indicated by the inhibition ratio was regulated to a lower level when the MFC was applied in a test of copper (II) [24]. To eliminate the signal interference between heavy metals, A. Khan et al., constructed a strain of *E. coli* (BL21), which was engineered to express the *znt*R, *rib*B, and *opr*F genes with the P_zntA_ promoter. The MFC sensor showed no response to Cu^2+^, Ni^2+^, Pb^2+^, Hg^2+^, and Co^2+^ metals in the detection of Zn^2+^, with a linear relationship of 0.9777 [25]. In another study, efforts were made to solve the signal interference between the organic matters and toxicity by utilizing a biocathode as a signal sensor, which was immune to the addition of acetate in the application of formaldehyde monitoring, but only the concentration of formaldehyde was monitored, and the signal of the acetate was ignored [17]. Despite all the research reported above, the monitoring of an individual analyte in a mixed solution by an MFC sensor has not been well considered or fully studied yet.

In view of this, we manipulated two MFC sensors, which were inoculated with sodium acetate (MFC-A) and glucose (MFC-B) separately, and a significant difference in the sensor sensitivities (the slope value of the linear-regression curve) was observed between MFC-A and MFC-B. The difference was then characterized and confirmed by the electrochemical characterization, CLSM, and microbial-community analysis. Herein, a novel hypothesis for the simultaneous test of sodium acetate and glucose in a mixed solution through the utilization of different sensor sensitivities is proposed. We assumed that the output voltages of MFC-A and MFC-B were in a linear relation with the concentrations of the individual analytes in the mixed solution, and afterwards, a system of linear equations of two variables (concentrations of two substances: sodium acetate and glucose) were formulated, and the solutions could then be calculated. The calculation results were compared to the true values, and satisfactory accuracies (relative error less than 20%) were obtained.

## 2. Materials and Methods

### 2.1. Construction and Operation of MFC Sensor

A dual-chamber MFC sensor (Phychemi Company Limited, Hong Kong, China) was constructed by cuboid organic glass blocks. The working volumes of the anode chamber and cathode chamber were both 50 mL. Two holes on the top of the electrode chamber were drilled for the feed injection and electrode connection. The electrode chambers were separated by a cation exchange membrane (Nafion 117, Dupont China Holding Co., Ltd., Beijing, China) before the MFC sensor was sealed with silicone gaskets. The cation exchange membrane was activated by a 30% hydrogen peroxide solution, and it was then cleaned and washed with 1 M sulfuric acid and deionized water. Graphite felt (diameter: 4 cm; thickness: 0.5 cm; Tianjin Carbon Co., Ltd., Tianjin, China) was applied as the anode, with an interval of 1 cm from the cathode (carbon fiber cloth; diameter: 4 cm; thickness: 0.2 cm; Tianjin Carbon Co., Ltd., Tianjin, China). Before usage, the graphite felt and carbon fiber cloth were immersed in 1 M sulfuric acid, 1 M sodium hydroxide, and acetone to remove the organic compounds. The electrodes were connected by a titanium wire with an external resistance of 1000 Ω. Domesticated sludge from the secondary clarifier of an incineration plant in Beijing was added into the anode chamber of the MFC sensor as the primary inoculum. The anolyte contained 0.5 g/L organic substrates (sodium acetate or glucose), 5 mL/L vitamins, and 12.5 mL/L trace minerals in a 50 mM phosphate buffer solution (PBS). The catholyte was potassium ferricyanide (100 mM potassium ferricyanide in 50 mM PBS). In the startup period, the MFC sensor was operated in batch mode at room temperature. The anolyte (nitrogen aerated for 10 min to maintain dissolved oxygen at less than 0.5 mg/L) and catholyte were refreshed after 24 h of inoculation. When the peak output voltage of five consecutive cycles remained stable, the startup of the MFC sensor was deemed successful. Two MFC sensors were started and acclimated in this study: MFC-A, with 0.5 g/L sodium acetate as the organic substrate, and MFC-B, with 0.5 g/L glucose as the organic substrate. Analytes with increased concentrations were also examined by the MFC sensors in different batches.

### 2.2. Analytical Methods

#### 2.2.1. Electrochemical Measurement

A data acquisition apparatus (EM9636M, BJZTLC technology Co., Ltd., Beijing, China) connected to a computer was utilized to collect the output voltage loading on the external resistance of the MFC sensor at an interval of 1 min. The current, current density, and power density of the MFC sensors were calculated according to Equations (1)–(3), respectively, where U is the observed output voltage, R is the external resistance, A presents the geometric surface area of the anode, and C_d_ and P_d_ are the current density and power density, respectively.

I = U/R (1)

C_d_ = I/A (2)

P_d_ = UI/A (3)

The power density and polarization curves indicated how the MFC sensor kept the voltage as a function of the generated current, and they were obtained by the drawing of the output voltage and power density against the current density [26]. They were tested when the output voltage of the MFC sensor was stable by varying the external resistance from 10 to 999,999 Ω. Coulombic efficiency (CE) represents the ration of generated current from the total COD reduction during the operation of MFC sensors, and it is calculated according to Equation (4):

CE = 100%(8C_T_M/FV△COD) (4)

where C_T_ is the total coulombs by the integrating current over time, F is the Faraday constant (96,485 C/mol), V represents the volume of the anode chamber, and △COD is the removed concentration of the COD [26].

An electrochemical workstation (DH7003, Jiangsu Donghua Analysis Instruments Co., Ltd., Taizhou, China) was applied to test the cyclic voltammetry (CV) and the electrochemical impedance spectroscopy (EIS) of the MFC sensor. The CV was recorded at room temperature by a three-electrode configuration with a saturated calomel electrode as the reference electrode in a PBS solution (0.1 M, pH = 7). The CV scan rate was 50 mV/s in the range from −0.6 to 0.8 V. EIS was carried out by the controlled potential method, with 10 mV amplitudes in a frequency range from 100,000 Hz to 0.01 Hz, at the condition of the open circuit potential.

#### 2.2.2. Anode Biofilm Analysis

Confocal laser scanning microscopy (CLSM) (Olympus FV1200, Olympus (China) Co., Ltd., Beijing, China) was applied to examine the live/dead cell distribution in the anode biofilm. SYTO-9 and propidium iodide (PI) were used to stain the live cells (green) and dead cells (red), respectively. The anode biofilms were washed in sterile 0.85% sodium chloride before and after the staining to clean the biofilms and remove the excess dye. When the output voltages were stable, the anode biofilms of MFC-A and MFC-B were collected for microbial-community analysis by 16S sRNA sequencing technology through the process of DNA extraction, PCR amplification, the fluorescent quantitative of the amplification products, and Illumina sequencing (Shiyanjia Lab Co., Ltd., Beijing, China).

## 3. Results and Discussion

### 3.1. Startup of MFC Sensors and Performance Characterization

**Figure 1:**
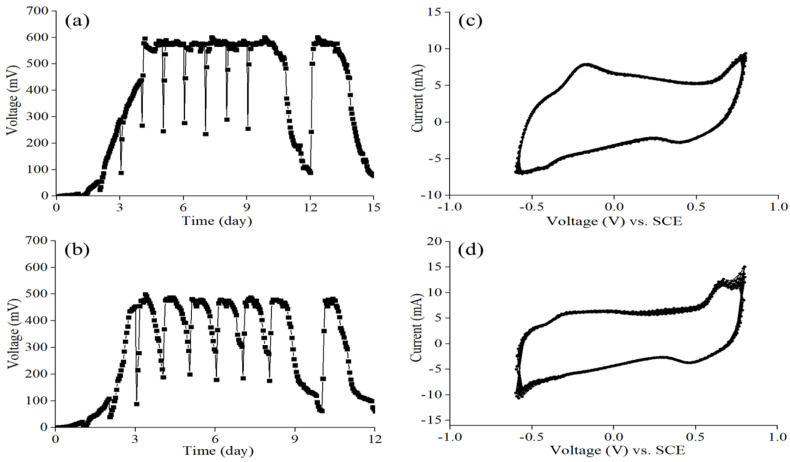
Output voltages of (**a**) MFC-A and (**b**) MFC-B. CV curves of (**c**) MFC-A and (**d**) MFC-B for 10 continuous cycles.

To explore the electrochemical performance of the MFC sensor, the output voltages of MFC-A and MFC-B, fed with 0.5 g/L sodium acetate and 0.5 g/L glucose, respectively, were monitored in the startup period. The batch mode was operated under closed circuit. As shown in Figure 1a, shortly after the operation of the MFC sensors, a gradual increase in the output voltage was observed. MFC-A delivered a stable output voltage after four cycles, and the maximum output voltage could reach 593.76 mV. As for MFC-B (Figure 1b), a stable output voltage with the maximum output voltage of 484.08 mV was obtained after three cycles. MFC-B showed a shorter startup period, as well as a lower output voltage, than MFC-A, and this could be due to the different substances in the anolytes, which have been confirmed to play an important role in the formation and maturation of biofilm, and to contribute significantly to the performance of MFC sensors. Moreover, the stable background output voltage of MFC sensors is essential for long-term monitoring [19]. Both MFC sensors showed a good stability of the electrochemical performance after the startup period, and the baseline of the maximum output voltage remained stable at around 590 mV of MFC-A (after four cycles), and 480 mV of MFC-B (after three cycles). Thus, a mature biofilm was believed to form on the surface area of the anode.

Besides the output voltage, the stability of MFC sensors could also be reflected by CV tests. The CV curves of MFC-A and MFC-B are shown in Figure 1c,d, and we can see that the currents of MFC-A and MFC-B were stable, with slight current losses after 10 continuous cycles. The results indicated that both MFC sensors possessed excellent stability and were suitable for applications as biosensors.

**Figure 2:**
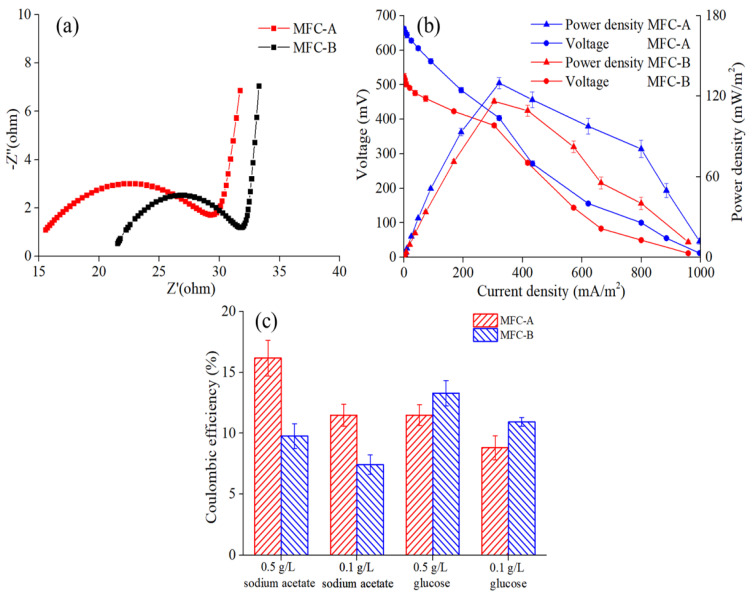
(**a**) EIS curves; (**b**) polarization curves of MFC-A and MFC-B; (**c**) CEs of MFC-A and MFC-B towards sodium acetate and glucose.

EIS, power density, and polarization curves were also applied for the evaluation of the bioelectricity performance of MFC sensors [1,27]. Both tests were conducted when the stable output voltage was achieved during the operation. The ohm resistance and charge transfer resistance could be deduced from the EIS curves, which indicated the capability of the electron transfer of the MFC sensors. As shown in Figure 2a, MFC-A and MFC-B delivered similar charge transfer resistances (13 and 12 Ω, respectively), which could be attributed to the favorable conductivity of anode materials. However, the ohm resistance of MFC-A was 16 Ω, which was much lower compared with the 21 Ω for MFC-B. This result indicated the higher electron transfer efficiency and higher degree of electrochemical activity of MFC-A. Figure 2b illustrates the power density and polarization curves of the MFC sensors. The intense limitation of kinetics in low current densities could be deduced as the voltage was reduced dramatically with the increase in the current density. MFC-A showed a superior power density compared with MFC-B under the same current density. The maximum power densities of MFC-A and MFC-B were 129.81 ± 4.10 and 116.21 ± 1.81 mW/m^2^, respectively, under an external resistance load of 1000 Ω. Open circuit voltage (OCV) has been reported to prove the electronic production capacity of MFCs, which could be influenced by electrode materials, bacteria, and so on [28]. The OCV of MFC-A was much higher than that of MFC-B (662.23 ± 5.83 mV vs. 523.04 ± 8.52 mV, respectively), and the result was consistent with the maximum power density mentioned above. This could be attributed to the organic substrates in the anolytes, which led to different communities of anode biofilms.

### 3.2. Individual Test of Sodium Acetate and Glucose

**Figure 3:**
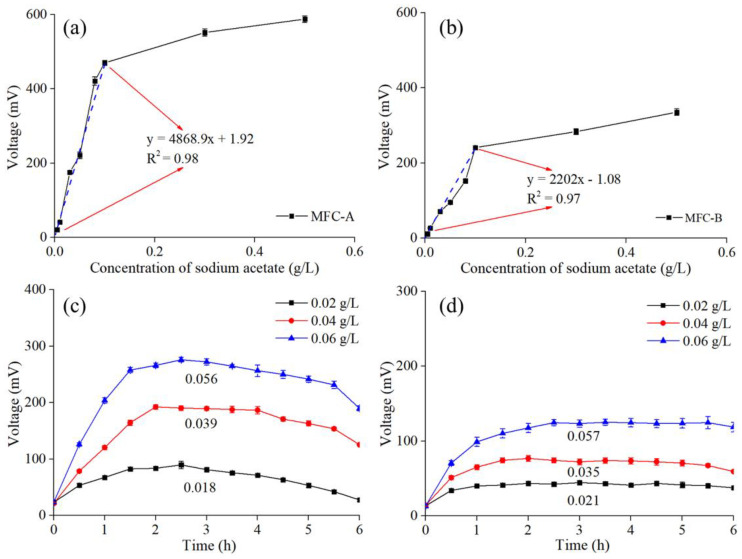
Linear-regression curves towards sodium acetate of (**a**) MFC-A and (**b**) MFC-B. Output voltages and calculated results of (**c**) MFC-A and (**d**) MFC-B during the sodium acetate test.

The concentration–response relationship is an important parameter of the monitoring sensitivity of an MFC sensor [19]. After the startup, the relationships of the MFC sensors between the analyte and output voltage were tested by varying the sodium acetate concentrations in the anolyte. The amendment of the sodium acetate concentrations led to an increase in the output voltages of MFC-A and MFC-B, in the range from 0.005 g/L to 0.1 g/L (Figure 3a,b). The sensitivities of the MFC sensors could be expressed by the output voltage signals per unit change in the concentration of the substances, and thus, the sensitivities of the MFC sensors were calculated as the slope values of the linear-regression curves [19]. The slope value of MFC-A was 4868.9 mV/(g/L sodium acetate), which was much higher than the 2202 mV/(g/L sodium acetate) of MFC-B. Moreover, significant linear relationships between the sodium acetate concentrations and maximum output voltages of the MFC sensors were observed (R^2^ of MFC-A = 0.98 and R^2^ of MFC-B = 0.97). These results indicated that MFC-A was more effective and sensitive than MFC-B. It was noticeable that the slope values of MFC-A and MFC-B dropped to 294.25 and 234.95 mV/(g/L sodium acetate), respectively, when the sodium acetate concentrations were higher than 0.1 g/L, and the linear-regression curves turned out to be smooth and no longer showed linear regression. To evaluate the accuracies of the MFC sensors, artificial solutions with 0.02, 0.04, and 0.06 g/L sodium acetate were tested by MFC-A and MFC-B. As shown in Figure 3c,d, the response times taken by MFC-A and MFC-B to reach the stable output voltage were approximately 1.5 and 2 h, respectively. The concentration value of the sodium acetate calculated from the output voltage and linear-regression curve was compared with the true value. Favorable accuracy rates were achieved by both MFC sensors. The accuracy rates of MFC-A were 90% (0.02 g/L sodium acetate), 97.5% (0.04 g/L sodium acetate), and 93.33% (0.06 g/L sodium acetate), while MFC-B had corresponding accuracy rates of 95%, 87.5%, and 95%, respectively.

**Figure 4:**
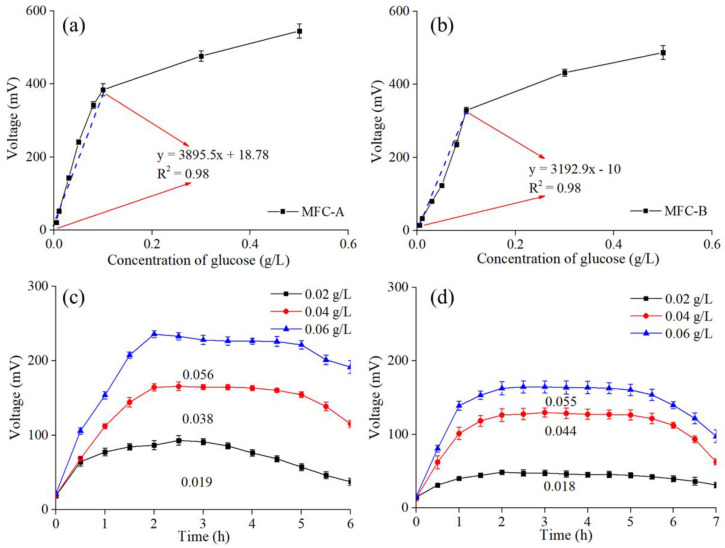
Linear-regression curves towards glucose of (**a**) MFC-A and (**b**) MFC-B. Output voltages and calculated results of (**c**) MFC-A and (**d**) MFC-B during the glucose test.

The sensitivities of MFC-A and MFC-B towards glucose were also investigated. And the correlation between the glucose and output voltage was found to be linear, as shown in Figure 4a,b. The detection of glucose examined in MFC-A and MFC-B showed that increasing concentrations of glucose led to an improvement in the output voltage. The linear-regression curve of MFC-A delivered a slope value of 3895.5 mV/(g/L glucose), which was higher than the 3192.9 mV/(g/L glucose) of MFC-B. Both MFC-A and MFC-B delivered a satisfactory regression coefficient of 0.98 when the glucose concentration was lower than 0.1 g/L. The results indicated that the output voltage alteration was directly related to the concentration of glucose. Moreover, the linear-regression curves showed a similar trend when the glucose concentration was higher than 0.1 g/L, where lower sensitivities were presented by MFC-A and MFC-B, with slope values of 402.73 and 395.72 mV/(g/L glucose), respectively. Artificial solutions of glucose were applied to evaluate the performances of the MFC sensors, and the results are shown in Figure 4c,d. The accuracy rates of MFC-A were 95% (0.02 g/L glucose), 95% (0.04 g/L glucose), and 93.33% (0.06 g/L glucose), while MFC-B had corresponding accuracy rates of 90%, 90%, and 91.67%, respectively.

To investigate the ability of exo-electrogenic bacteria in anode biofilm, the CEs of MFC-A and MFC-B towards sodium acetate and glucose were calculated. As shown in Figure 2c, MFC-A achieved higher CEs towards sodium acetate (16.17 ± 1.47% of 0.5 g/L and 11.48 ± 0.89% of 0.1 g/L) than those of MFC-B (9.76 ± 1.02% of 0.5 g/L and 7.41 ± 0.80% of 0.1 g/L). This indicated that the electronic-generation ability of MFC-A was higher than that of MFC-B in the metabolism of sodium acetate. However, MFC-B obtained higher CEs towards glucose (13.29 ± 1.04% of 0.5 g/L and 10.93 ± 0.36% of 0.1 g/L) than those of MFC-A (11.49 ± 0.86% of 0.5 g/L and 8.81 ± 0.99% of 0.1 g/L). Moreover, the CEs were observed to decrease with the decrease in the organic substrate’s concentration, and this could be attributed to the fact that the organics were mostly consumed for bacterial growth rather than electricity generation.

The above results demonstrated that organic substrates in anolytes exerted a significant influence on the performance of MFC sensors, and MFC-A displayed a superior sensitivity to that of MFC-B for both sodium acetate and glucose. This could be attributed to the difference in the anode biofilms caused by organic substrates. The obtained results in this study were in accordance with the previous reports that found that the substrate concentration, anode area, and external resistance could affect the sensitivities of MFC sensors [13]. The results of the CEs also demonstrated the different metabolic capabilities between MFC-A and MFC-B.

### 3.3. Simultaneous Test of Sodium Acetate and Glucose in a Mixed Solution

Many attempts have been made to explore MFC sensors, but the attention has mainly been focused on the detection of mixed solutions with respect to the COD or toxicity [14,29]. The simultaneous test of individual substances in a mixed solution has not been fully studied. Based on the results obtained above, a simultaneous test of sodium acetate and glucose in a mixed solution by MFC sensors was carried out and evaluated.

To evaluate the performance of the MFC sensors for the simultaneous test of analytes in a mixture, three mixed solutions were prepared and applied as anolytes of MFC-A and MFC-B (i.e., S1 (0.01 g/L sodium acetate and 0.01 g/L glucose); S2 (0.02 g/L sodium acetate and 0.02 g/L glucose); S3 (0.03 g/L sodium acetate and 0.03 g/L glucose)). The stable output voltages of MFC-A and MFC-B generated with the three mixed solutions were achieved in 2 and 3 h, respectively, and much higher output voltages were delivered by MFC-A (S1: 121.98 mV; S2: 218.39 mV; S3: 306.03 mV) than MFC-B (S1: 50.47 mV; S2: 109.81 mV; S3: 163.76 mV). Based on the hypothesis that the output voltage generated from the mixed solution equaled the sum of that generated by the individual solute sodium acetate and glucose, the concentrations of sodium acetate and glucose could be calculated according to Equations (5) and (6), respectively (deduced from the linear-regression curves of Figure 3a,b and Figure 4a,b, respectively). V_A_ and V_B_ are the output voltages of MFC-A and MFC-B, while x and y represent the concentrations of sodium acetate and glucose, respectively. The calculation results of the three solutions are as follows: sodium acetate: 0.012 g/L and glucose: 0.011 g/L of S1; sodium acetate: 0.023 g/L and glucose: 0.022 g/L of S2; sodium acetate: 0.033 g/L and glucose: 0.032 g/L of S3. Relative errors from 6.7% to 20% (sodium acetate: 20% and glucose: 10% of S1; sodium acetate: 15% and glucose: 10% of S2; sodium acetate: 10% and glucose: 6.7% of S3) were achieved, which proved that the proposal is feasible for the simultaneous test of sodium acetate and glucose in a mixture.

V_A_ = 4869x + 3895.5y + 20.7 (5)

V_B_ = 2202x + 3192.9y − 11.1 (6)

According to the hypothesis of our study, a new light was shed on the problem of signal interference by the setup of the MFC-sensor groups with different sensitivities to toxicity or the COD. For example, chemical toxicants have a negative effect on the metabolic activity of microorganisms, and they hinder the transfer rate of electrons to the electrode. Thus, the presence of chemical-toxicant samples with totally different CODs may present similar values through MFC sensors [16]. By utilizing the different sensitivities, this problem (especially between toxics and organic substances) could be timely noticed and avoided. Moreover, a higher COD was reported to restrict the electroactivity of an MFC sensor, and so the accuracy of MFC sensors could be further compromised [30]. In view of this, methods applied in this research could be employed to avoid the fake signals during the operation of MFC sensors.

### 3.4. Analysis of Anode Biofilm

**Figure 5:**
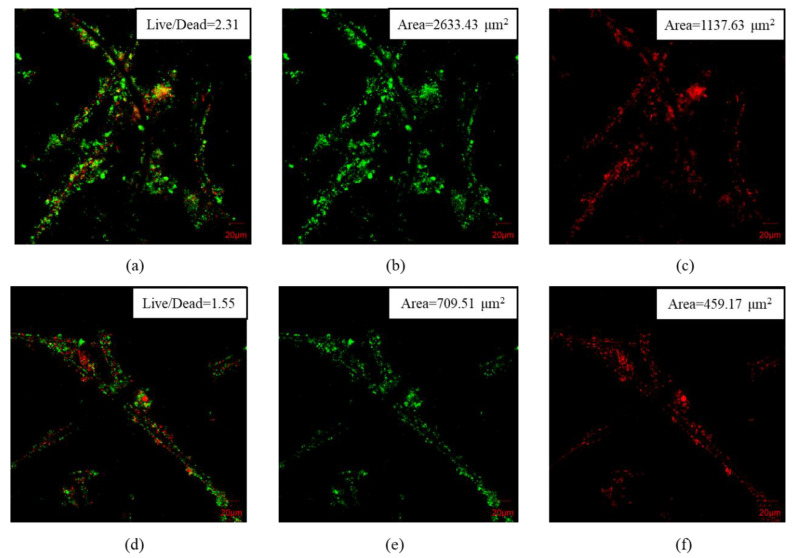
CLSM images of anode biofilm of MFC-A: (**a**) merged picture of living and dead cells; (**b**) picture of live cells; (**c**) picture of dead cells and MFC-B: (**d**) merged picture of living and dead cells; (**e**) picture of live cells; (**f**) picture of dead cells. Live cells: green; dead cells: red.

The anode biofilm plays an important role in the performance of MFC sensors, as it directly interacts with organic substrates and delivers a responding output signal [31]. The properties of the anode biofilm could be affected by the materials of the anode, organic substrates in the electrolytes, the operation parameters, and so on, thereby influencing the electrochemical performance, as well as the sensitivities of MFC sensors [19,32,33]. In this study, CLSM was applied to semi quantitively analyze the spatial distribution of bacteria in an anode biofilm [19]. As presented in Figure 5, the CLSM images of the anode biofilms clearly illustrate that MFC-A developed a richer biofilm with more bacteria than MFC-B. A richer biofilm has been reported to further enhance the interface between the analytes and bacteria, thus boosting the mass transfer rate and improving the monitoring performance of MFC sensors [34]. Moreover, more viable bacteria were observed near the biofilm surface of MFC-A, which led to a higher degree of electrochemical activity [35]. As shown in Figure 5b,e, an obvious distinction was observed between MFC-A and MFC-B. The live cell area (analyzed by software: Image J, National Institutes of Health, Bethesda, United States) of MFC-A was 2633.43 μm^2^, which was 271.16% higher than that of MFC-B (709.51 μm^2^). Besides, difference in the amount of dead cell between two MFC sensors were also obvious in accordance with the live cell as shown in Figure 5c,f. Another obvious distinction was that the live/dead cell ratio of MFC-A was 2.31, which was also much higher than the 1.55 of MFC-B, as shown in the CLSM images (Figure 5a,d). In one word, the CLSM results above indicated that the biofilm has a positive effect on the electrochemical activity of MFC sensors, and MFC sensors with richer biofilms and higher live/dead cell ratios are supposed to possess superior monitoring performances.

**Figure 6:**
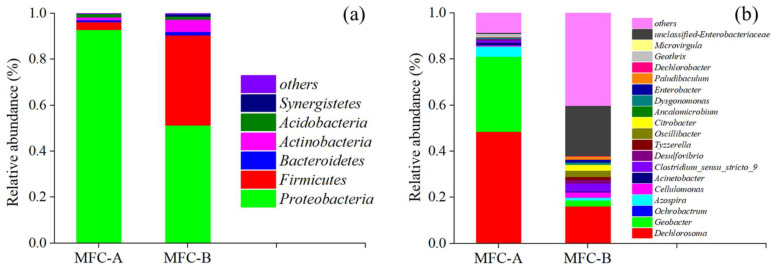
Relative abundances of anode microbial community by 16 S rRNA gene sequencing at the level of (**a**) phylum and (**b**) genus.

The microbial communities of the anode biofilms were also characterized, and an obvious difference was observed between MFC-A and MFC-B. On the phylum level (Figure 6a), sodium acetate in the anolyte increased the relative abundance of *Proteobacteria*, which was more dominant in the anode biofilms of MFC-A (92.90%) than those of MFC-B (51.33%). This indicated a superior selectivity of sodium acetate compared with glucose. The Shannon indexes of MFC-A and MFC-B were 3.81 and 5.51 respectively, which also reflected the higher species selectivity of MFC-A. Moreover, the *Firmicutes* of MFC-B demonstrated a relative abundance of 39.15%, which was much higher than that of MFC-A (3.49%), and this is probably due to the metabolism of the glucose in MFC-B. As we know, *Proteobacteria* and *Firmicutes* are considered as the key composition of electroactive biofilms. The relative abundance of *Proteobacteria* and *Firmicutes* in MFC-A and MFC-B were both higher than 90%, confirming a mature electroactive biofilm [36]. Similar results could also be observed on the genus level, as shown in Figure 6b. The predominant bacteria of MFC-A were *Dechlorosoma* and *Geobacter*, with a relative abundance of 81.11%. Previous research has reported *Geobacter* to possess a dominant relative abundance (35.22%) as electroactive bacteria in anode biofilms [19]. *Dechlorosoma* was the predominant bacteria in the anode biofilm of MFC-B, with a relative abundance of 16.16%. To conclude, the results of the microbial-community analysis at the phylum and genus levels were consistent with the electrochemical and monitoring performances of MFC sensors.

It is worth mentioning that the microbial community could vary after the long-term utilization of MFC sensors. Although a short exposure time was proven to insignificantly change the microbial community of biofilm [19], the regular evaluation of the MFC sensor sensitivity, as well as the linear-regression curve, is important in the application of MFC sensors for accurate results.

## 4. Conclusions

The main issues addressed in this paper are as follows. (a) Two MFC sensors fed with sodium acetate and glucose were developed, and different electrochemical performances between the two MFCs were observed and evaluated. (b) Different monitoring performances of both MFC-A and MFC-B towards sodium acetate and glucose were confirmed, illustrated by the slope values of the linear-regression curves. (c) According to the different sensitivities, an innovative method for the simultaneous test of sodium acetate and glucose in a mixed solution was proposed. The linear equations of two unknowns (concentrations of sodium acetate and glucose) were formulated, and the solutions showed the desired accuracy (relative error less than 20%). (d) Our research sheds new light on the application of MFC sensors for the simultaneous monitoring of individual analytes in a mixture.

## Data Availability

The data used to support the findings of this study are available from the corresponding author upon request.
